# Polymorphisms in the Inflammatory Genes *CIITA*, *CLEC16A* and *IFNG* Influence BMD, Bone Loss and Fracture in Elderly Women

**DOI:** 10.1371/journal.pone.0047964

**Published:** 2012-10-25

**Authors:** Maria Swanberg, Fiona E. McGuigan, Kaisa K. Ivaska, Paul Gerdhem, Kristina Åkesson

**Affiliations:** 1 Clinical and Molecular Osteoporosis Research Unit, Department of Clinical Sciences, Lund University, Sweden; 2 Neurodegeneration and Inflammation Genetics Unit, Department of Experimental Medical Science, Lund University, Sweden; 3 Department of Cell Biology and Anatomy, Institute of Biomedicine, University of Turku, Turku, Finland; 4 Department of Clinical Science, Intervention and Technology, Karolinska Institute, Stockholm, Sweden; 5 Department of Orthopaedics, Karolinska University Hospital, Stockholm, Sweden; 6 Department of Orthopaedics, Skåne University Hospital, Malmö, Sweden; University of Hong Kong, Hong Kong

## Abstract

Osteoclast activity and the fine balance between bone formation and resorption is affected by inflammatory factors such as cytokines and T lymphocyte activity, mediated by major histocompatibility complex (MHC) molecules, in turn regulated by the MHC class II transactivator (MHC2TA). We investigated the effect of functional polymorphisms in the MHC2TA gene (*CIITA*), and two additional genes; C-type lectin domain 16A (*CLEC16A)*, in linkage disequilibrium with *CIITA* and Interferon-γ (*IFNG*), an inducer of *CIITA;* on bone density, bone resorption markers, bone loss and fracture risk in 75 year-old women followed for up to 10 years (OPRA n = 1003) and in young adult women (PEAK-25 n = 999). *CIITA* was associated with BMD at age 75 (lumbar spine p = 0.011; femoral neck (FN) p = 0.049) and age 80 (total body p = 0.015; total hip p = 0.042; FN p = 0.028). Carriers of the *CIITA* rs3087456(G) allele had 1.8–3.4% higher BMD and displayed increased rate of bone loss between age 75 and 80 (FN p = 0.013; total hip p = 0.030; total body p = 3.8E^−5^). Despite increasing bone loss, the rs3087456(G) allele was protective against incident fracture overall (p = 0.002), osteoporotic fracture and hip fracture. Carriers of *CLEC16A* and *IFNG* variant alleles had lower BMD (p<0.05) and ultrasound parameters and a lower risk of incident fracture (*CLEC16A,* p = 0.011). In 25-year old women, none of the genes were associated with BMD. In conclusion, variation in inflammatory genes *CIITA*, *CLEC-16A* and *INFG* appear to contribute to bone phenotypes in elderly women and suggest a role for low-grade inflammation and MHC class II expression for osteoporosis pathogenesis.

## Introduction

Osteoporosis is a common disease in our ageing society and affects one in three women during the course of their lifetime. The disease is characterized by quantitative and qualitative changes to bone tissue and results in increased risk of fractures [1]. The etiology of osteoporosis is complex and contains both genetic and environmental risk factors, as well as interactions between them. As much as 80% of the population variance in bone mineral density (BMD) is genetically determined and results from many common alleles conferring small risk increments [2]. Genes may be phenotype specific or pleiotropic within the context of osteoporosis or shared with other complex diseases [2], [3].

The role of the immune system is well established in conditions that are primarily inflammatory such as rheumatoid arthritis (RA), systemic lupus erythematosus (SLE) and multiple sclerosis (MS), however, systemic inflammatory processes are involved in other complex diseases such as obesity, diabetes and cardiovascular disease, and possibly also osteoporosis [4]–[6]. These diseases are generally more prevalent with increasing age and shared common risk factors have been identified [5], [6]. While patients with some chronic inflammatory disorders are more likely to develop osteopenia, osteoimmunological interplay also constitutes part of normal physiological processes in bone.

The interplay between the immune system and bone metabolism includes molecular and cellular interactions between haematopoietic cells, lymphocytes, osteoblasts and osteoclasts, which are derived from the monocyte-macrophage lineage [2], [3]. These interactions involve major histocompatibility complex (MHC) molecules and cytokines that have dual roles in bone homeostasis [3], [7], [8]. Antigen presentation on MHC molecules and the resulting activating patterns are crucial to the degree and type of immune response. Macrophage lineage cells are thus capable of both eliciting and modulating the inflammatory response by presenting MHCII molecules and activating T lymphocytes. The MHCII transactivator (MHC2TA) assembles transcription factors at promoter sites and is the master regulator of MHCII expression [9]. Expression of MHC2TA is crucial for proper antigen presentation [10] and is in turn induced by the cytokine interferon-γ (IFNγ).

Allelic variation in MHCII molecules contributes to several diseases including MS [11], RA [12], [13] and osteoarthritis [14], [15] while Genome-wide association studies (GWAS) of bone density and fracture have identified the human leukocyte antigen (HLA) region on chromosome 6p21, which encodes many immune-related genes including MHCII [16], [17]. In the HLA region, the common rs3130340(T) allele was found to be associated with reduced BMD at the spine and increased risk of low trauma fractures [17]. Combining GWAS and gene expression data, Farber performed weighted gene co-expression network analysis (WGCNA) and identified a co-expression module highly enriched for genes involved in immune processes [18]. In addition, association studies have identified the pro-inflammatory interleukin 6 (IL6) gene as a determinant for low BMD [19]–[22], while in a previous study of the pro-inflammatory cytokine macrophage migration inhibitory factor (*MIF*) gene we reported association of *MIF* polymorphisms with bone loss and increased levels of bone resorption markers in elderly women [23].

A more pro-inflammatory profile characterized by increased levels of IFNγ and changes in cytokine regulation is evident in perimenopausal women and may induce bone loss. For example, activated T lymphocytes produce tumor necrosis factor-α (TNFα), an instrumental cytokine contributing to increased osteoclast activity and subsequent loss of bone density [24]. Studies in mice report a key role for MHC2TA in ovariectomy-induced bone loss, where the increased production of IFNγ increased MHC2TA expression, leading to lymphocyte activation and production of TNFα [25]. Based on the link between bone and inflammation, we hypothesize that polymorphisms of genes in the IFNγ-MHC2TA-MHCII pathway have implications for susceptibility to postmenopausal and senile osteoporosis. To date, this has not been explored as a determinant of bone loss and fracture with advancing age or at the time of maximal bone mass. In this study, we therefore analyzed polymorphisms in three genes encoding proteins regulating adaptive immune responses; MHC2TA (*CIITA*; 16p13), C-type lectin domain 16A (*CLEC16A*; 16p13) and IFNγ (*IFNG*; 12q14).

The rational for selecting these genes for analysis is based on considerable evidence. For example, null mutations in *CIITA* cause severe immunodeficiency [10] while common functional polymorphisms in the gene affect MHCII expression levels and susceptibility to inflammatory and complex diseases [26]–[33]. *CLEC16A* is located adjacent to and in linkage disequilibrium (LD) with *CIITA*, motivating analysis of this gene in conjunction with *CIITA*. Furthermore, CLECs play important roles in adaptive immunity by shaping the cytokine profile [34] and associations between *CLEC16A*, both in conjunction and independent of *CIITA* have been reported in several inflammatory conditions [31], [35]–[38]. IFNγ induces MHCII expression through *CIITA* and polymorphisms in *IFNG* are associated with differential gene expression and disease susceptibility [39]. In postmenopausal women, the production of pro-inflammatory cytokines such as IFNγ is increased [40] and studies in ovariectomized mice have shown increased production of IFNγ, enhanced activation of T lymphocytes through MHC2TA and increased bone loss [25].

To address the genetic impact of polymorphisms in genes with possible effects on osteoimmunological interactions involving MHCII, T-lymphocytes and osteoclast activation, we studied both young and elderly women; 1003 75-year old women followed for up to 10 years and 999 25-year old women in order to capture age-related differences on BMD, bone loss and incident fracture risk.

## Methods

### Study Subjects

The Malmö Osteoporotic Prospective Risk Assessment (OPRA) cohort is a longitudinal population-based cohort of Caucasian women aged 75 years who were randomly selected from the Malmö city files between 1995 and 1999. No exclusion criteria were applied. A total of 1604 women were invited, 1044 (65%) attended at baseline and 715 women completed the 5 year follow-up visit.

In this study, BMD was measured at baseline and 5 years, with additional measurements available in 371 women at 10 years for hip and total body. Bone resorption markers were measured in serum at baseline and at the 1-, 3-, and 5-year follow-up visits. Information on medication, smoking and illness was collected by a questionnaire. Of the 1044 women at baseline, 145 (14%) were smokers, 208 (20%) were former smokers, 69 had diabetes, 48 were using hormone replacement therapy (HRT) or bisphosphonates at baseline (119 were using one or both of these medications at baseline or sometime during the 5 year follow-up period). Full details of this cohort have been reported previously [41].

The PEAK-25 cohort is a population-based cohort of Caucasian women aged 25 years living in Malmö. Initially, 2394 women randomly selected from the city files between 1999 and 2004 were invited, 1059 (44%) attended the full investigation. The exclusion criteria used were pregnancy or recent childbirth (n = 102, 7.6% of non-participants). Of those who participated, 56% were non-smokers 26% were current smokers and 18% were previous smokers. Full details of this cohort have been reported previously [42].

All participants gave written informed consent and the Lund University Ethics Committee approved the study. This study was performed according to the principles of the Helsinki declaration.

The data reported in this analysis is based on women for whom genotype and phenotype data was available, corresponding to 1003 women from the OPRA cohort at baseline and 999 women from the PEAK-25 cohort. Clinical characteristics of the women attending the baseline visit are shown in Table 1.

**Table 1:** Baseline clinical characteristics of the OPRA and PEAK-25 cohorts.

	OPRA (n = 1003)			PEAK-25 (n = 999)		
Variable	Mean	±SD	(Range)	Mean	±SD	(Range)
Age (years)	75.2	0.1	(75.0−75.9)	25.5	0.2	(25.0−25.9)
Weight (kg)	67.8	11.5	(41−110)	64.6	11.1	(40−135)
Height^a^ (cm)	164.1	5.5	(145−180)	167.6	6.1	(150−187)
**BMD (g/cm^2^)** b						
Total body	1.007	0.097	(0.718−1.422)	1.174	0.073	(0.969−1.478)
Femoral neck	0.748	0.130	(0.153−1.230)	1.055	0.124	(0.746−1.604)
Total hip	0.849	0.149	(0.498−1.416)			
Lumbar spine	0.993	0.195	(0.518−1.855)	1.217	0.129	(0.824−1.868)
**Ultrasound** c						
BUA	102	10	(56−136)	118	11	(59−149)
SoS	1523	27	(1425−1643)	1575	32	(1498−1706)
Stiffness	72	13	(31−112)	99	15	(42−150)

a Height at age 20 (OPRA), Height at baseline visit, age 25 (PEAK25).

b BMD n = 904–946 (OPRA), n = 996–999 (PEAK-25).

c QUS n = 853 (OPRA), n = 853 (PEAK-25).

### Marker Selection

Polymorphisms in *CIITA*, *CLEC16A* and *INFG* were selected based on previously reported associations with inflammatory disorders, other diseases or differential gene expression [29]–[31], [33], [35]–[37], [39], [43], [44]. The SNPs have not previously been identified in GWAS of osteoporosis related traits. Marker details and criteria for selection are shown in Table 2.

**Table 2:** Markers used in the study and allele frequency in the OPRA cohort.

SNP ID	LocationChr (bp)	Common allele (frequency)	Minorallele (frequency)	Selection criteria
***CIITA***				
rs3087456	16 (10970902)	A (0.765)	G (0.235)	Association with inflammatory disease and differential gene expression.[29], [30], [33]
rs4774	16 (11000848)	G (0.689)	C (0.311)	
***CLEC16A***				
rs725613	16 (11169683)	T (0.660)	G (0.340)	Associated with type 1 diabetes and multiple sclerosis.[31], [35], [73]–[75]
rs2903692	16 (11238783)	G (0.678)	A (0.322)	
rs6498169	16 (11249329)	A (0.604)	G (0.396)	Associated with multiple sclerosis and rheumatoid arthritis[31], [36], [76]
***IFNG***				
rs2069727	12 (68548223)	T (0.532)	C (0.468)	Located 3′ near gene
rs2069718	12 (68550162)	C (0.582)	T (0.418)	Association with SLE. Located in intron 3 [39]
rs2069705	12 (68555011)	T (0.626)	C (0.374)	Association with SLE. Located 5′ upstream of gene [39]

### Genotyping

Total genomic DNA was isolated from blood using the QIAamp 96 DNA blood kit (Qiagen, Valencia, CA, USA) according to the manufacturer’s instructions.

Genotyping of *CLEC16A* (rs6498169) and *INFG* polymorphisms rs2069705 and rs2069727 was performed using Taqman SNP genotyping Assay (Applied Biosystems, Foster City, CA, USA). PCR was conducted in a Dual 384-well GeneAmp PCR system 9700 (Applied Biosystems), with an endpoint plate read on ABI 7900HT (Appied Biosystems) using the SDS 2.2.2 software. Polymorphisms for *CIITA* (rs3087456 and rs4774), *CLEC16A* (rs2903692 and rs725613) and *INFG* (rs2069718) were genotyped by Sequenom’s iPlex Gold system (Sequenom, San Diego, CA). The overall success rate for both methods was >98 for all polymorphisms. Approximately 3% of the samples from each cohort were genotyped in duplicate with 100 percent concordance. All polymorphisms conformed to Hardy-Weinberg equilibrium. Allele and genotype frequencies are reported in Supplementary Table S1.

### Bone Density

The areal BMD was measured with dual-energy x-ray absorptiometry (DXA) by Lunar DPX-L for OPRA and Lunar Prodigy for PEAK-25 (Lunar Corporation WI, USA and GE Health Lunar Densitometry, WI, USA) at all sites (total body (TB), femoral neck (FN), total hip (TH) and lumbar spine L2–L4 (LS)). Analyses of OPRA scans were made with software versions 1.33 and 1.35 at baseline, 4.7b at five years and 4.7e at 10 years. The total hip scans were all analyzed with version 4.7b. There was no drift in phantom measurement results during the study period. Analyses of PEAK-25 scans were made with software versions 2.05, 2.15, 3.60, 5.70 and 7.70. DXA calibrations were performed daily using a manufacturer supplied phantom and the precision error (coefficient of variation) was total body; 0.94, LS; 1.45 and FN; 4.01 [45].

The rate of bone loss (RBL) between age 75 and 80 represents the annual change in BMD between the scan at baseline (BMD_BL_) and the 5-year follow-up (BMD_5Y_). It was calculated as [(BMD_5Y_-BMD_BL_)/BMD_BL_/years between scans*100]. The LS BMD at age 80 and LS RBL are not reported since previously we showed that LS BMD increased likely due to the presence of osteophytes, degenerative changes and compressive fractures common in women of this age [46]. Weight and BMD at age 75, 80 and 85 along with RBL is presented in Supplementary Table S3.

### Quantitative Ultrasound

Quantitative ultrasound measurement speed of sound (SoS), broadband ultrasound attenuation (BUA) and stiffness index (SI, derived from of BUA and SoS) were assessed in both cohorts using a Lunar Achilles® system (Lunar Corporation, Madison, WI, USA). The right calcaneus was measured unless precluded by a previous history of injury or fracture. Precision was 1.5 for BUA and SOS [47] and calibrations were performed daily.

### Fracture

Fractures in the OPRA cohort were self-reported for adult fractures (age 20–75) at the time of inclusion in the study and were verified from radiological files as previously reported [48]. In addition, incident fractures during the follow-up period (until November 2006) were recorded and verified by reviewing the related medical records [49]. The majority of fractures sustained (>99%) were attributable to low energy trauma. Fractures were classified as being osteoporotic if affecting the hip, distal radius, spine or proximal humerus.

### Biochemistry

The serum bone markers C-terminal cross-linking telopeptide of type I collagen (S-CTX) and serum tartrate-resistant acid phosphatase 5b (S-TRACP5b) were analyzed in the OPRA cohort. S-TRACP5b was determined with BoneTRAP® assay (SBA Sciences/Immunodiagnostic Systems IDS Inc., Bolton, UK) and S-CTX was determined with Elecsys β-CrossLaps immunoassay (Roche Diagnostics, Indianapolis, IN). Details of the assays have been reported previously [46].

Longitudinal measures of bone resorption markers at baseline, 1-, 3-, and 5-years (age 80) were used. Among the 1003 women included in this study, data for all time points was available from 614 subjects for S-TRACP5b and from 606 subjects for S-CTX. Subgroups were created according to whether women had constantly low or constantly or high levels of S-CTX or S-TRACP5b over 5 years, as described previously. Those in the lowest or highest tertiles at three or more time points were considered to have constantly low or high levels, respectively, over the 5-year period. All others were classified into the intermediate group [50]. Among the 1003 women included in this study, 93 were constantly high and 108 constantly low for S-TRACP5b and 121 constantly high and 117 constantly low for S-CTX. Women using potent estrogen or bisphosphonates were excluded from the analyses (S-TRACP5b n = 76, S-CTX n = 77) since these medications have been shown to decrease S-CTX levels [51], [52].

Routine biochemistry measured C-reactive protein (CRP, mg/L) in plasma, erythrocyte sedimentation rate (ESR, mm/h) in blood and leukocytes (BLC 10^9^/L) in blood at 10-year follow-up.

### Statistics

Deviation from Hardy-Weinberg equilibrium was calculated by the chi-square test. Linkage disequilibrium (LD) was evaluated within each gene and between *CIITA* and *CLEC-16A* using the Haploview program (http://www.broad.mit.edu/mpg/haploview/) and taking into consideration both D and r^2^. Haplotypes were constructed for *CLEC16A* and *IFNG* using PHASE version 2.02 [53], and estimated haplotypes with a frequency >0.10 and a >0.8 probability of correct assignment were used for analysis.

ANOVA was used to determine associations between genotypes, bone density and rate of bone loss.

Bone resorption marker values were log-transformed prior to analysis due to non-normal distribution. The t-test was used for comparing mean S-CTX and S-TRACP5b values, and the Pearson Chi-Square test was used for analyzing consistently high and low levels of markers and association of polymorphisms with fracture incidence.

Linear regression was used to identify confounding factors for BMD, RBL and ultrasound and where indicated, multinomial logistic regression was used to adjust for total body BMD and weight at baseline in fracture analyses. Age was not included for adjustment since all the women within each cohort were of the same age.

The following covariates were used in the analyses of the OPRA cohort. BMD at baseline: weight (baseline), diabetes, current use of bisphosphonates (BPs) or potent estrogen (E) and smoking exposure (cigarettes per day*years smoking); BMD at 5-year follow-up: weight (5-year follow-up) and smoking exposure; BMD at 10-year follow-up: weight (10-year follow-up); RBL: use of E and/or BPs at baseline or during follow-up; QUS: weight (baseline), current use of BPs or E and smoking exposure. Exclusion of estrogen or bisphosphonate users from the analysis (n = 82) did not appreciably alter the results overall.

When indicated, weight change between baseline and 5-year follow-up visit was also used as covariate for RBL. Covariates used in the PEAK-25 cohort were height, weight and smoking for BMD; weight and smoking for ultrasound phenotypes.

Interaction between polymorphisms may contribute to opposite or synergistic effects on the phenotype of interest. To this end we performed pairwise interactions between SNPs to explore the effect on differences in BMD and QUS. Using general linear model-ANOVA (GLM-ANOVA), the model included an interaction term, covariates and assumed a co-dominant genetic model. In the results, p-values are reported for the interaction overall while in the tables mean BMD values defined by SNP_1_-SNP_2_ carriers compared to SNP_1_-SNP_2_ non-carriers are presented.

*A priori* power analyses indicated that the sample size of our studies allowed a >80% power to detect a difference in BMD of 0.1 standard deviations among genotype groups, while maintaining a type I error at 5%. This is based on the assumption of a SD of 0.13 g/cm^2^ in BMD, which allows detection of a 0.065 g/cm^2^ difference between genotype groups assuming a minor allele frequency of 0.21 or greater. Similarly, the OPRA study had >80% power to detect relative risks for fracture of at least 1.2.

All statistical analyses were performed using SPSS for Windows 18.0 (SPSS Inc., Chicago, IL) and associations with a p-value of <0.05 were considered nominally significant. The phenotypes and several of the markers studied are not independent (i.e. are correlated or in LD), therefore applying a Bonferroni correction would be over-stringent. We therefore report the uncorrected p-values and acknowledge the fact that multiple tests were performed.

## Results

### CIITA

The 2 SNPs in *CIITA* were not in strong LD with each other (D’<0.36, r^2^<0.02) or with the 3 SNPs in the adjacent *CLEC16A* gene (D’<0.45, r^2^<0.04). Haplotype estimates predicted 3 common (>10%) haplotypes (Supplementary Table S2). Haplotype analysis for *CIITA* did not add appreciably to the overall information obtained from genotype analyses (data not shown).

### Bone Density and Bone Loss

In the OPRA cohort of elderly women, there was a moderate association between *CIITA* and BMD. Carriers of the rare rs3087456(G) allele displayed higher FN BMD at age 75 (baseline) and interaction between variant alleles rs3087456(G) and rs4774(C) was observed, resulting in higher LS BMD. At age 80 (5-year follow-up), FN BMD and TB BMD were also higher in rs3087456(G) carriers and an interaction between rs3087456(G) and rs4774(C) was associated with higher BMD at all sites (Table 3). At 10-year follow-up (n = 376), interaction between rs3087456(G) and rs4774(C) variant alleles was observed, with higher BMD at the FN (p = 0.019) TH (p = 0.027) and TB (p = 0.004) compared to common homozygotes (rs3087456(G): FN +0.4%, TH +1.7%, TB +1.8%; rs4774(C): FN +1.9%, TH +2.5%, TB +1.5%), data not shown.

**Table 3:** Associations between BMD and *CIITA*, *CLEC16A* and *IFNG* in the OPRA cohort.

Gene	SNP (rare allele)/haplotype	Site	Age	BMD mean^1^ (g/cm^2^)	P-value*
***CIITA***	rs3087456(G)	**FN**	75	0.740/0.761	0.049
	rs3087456(G)*rs4774(C)	**LS**	75	0.967/0.992	0.011^2^
	rs3087456(G)	**FN**	80	0.702/0.726	0.041
	rs3087456(G)*rs4774(C)	**FN**	80	0.692/0.717	0.020^3^
	rs3087456(G)*rs4774(C)	**TH**	80	0.784/0.803	0.042
	rs3087456(G)	**TB**	80	0.989/1.009	0.015
	rs3087456(G)*rs4774(C)	**TB**	80	0.981/1.005	0.027
***CLEC16A***	Haplotype 1^4^	**LS**	75	1.002/0.983	0.037
	Haplotype 1^4^	**FN**	80	0.716/0.706	0.035
	Haplotype 1^4^	**TH**	80	0.802/0.794	0.039
	rs6498169	**TB**	80	0.997/1.002/0.985	0.037
***IFNG***	Haplotype 4^5^	**TH**	75	0.854/0.846	0.035
	rs2069727*rs2069705	**LS**	75	0.973/1.007/0.9890.993/1.002/0.962	0.027

* Only associations where p<0.05 are reported.

1 BMD values are reported as follows: For single SNP analysis (non-carrriers/carriers or common homozygotes/heterozygotes/variant homozygotes); For haplotypes (non-carriers/carriers); For interactions in allelic models (non-carriers of both variant alleles/carriers of both variant alleles); For interactions in genotypic models, values are given for each marker as common homozygotes/heterozygotes/variant homozygotes since no individuals were carriers of both variant alleles.

2 For rs3087456 * rs4774 genotypic model, p = 0.011.

3 For rs3087456 * rs4774 genotypic model, p = 0.028.

4 CLEC16A haplotype 1: rs725613(T)/rs2903692(G)/rs6498169(A).

5 IFNG haplotype 4: rs2069727(T)/rs2069718(T)/rs2069705(C).

The QUS results were in line with those for BMD with rs3087456(G) carriers having higher mean values for stiffness (72.9/70.6; p = 0.030) and BUA (102.6/101.1; p = 0.045).

Homozygotes for the rare *CIITA* allele rs3087456(G) had higher annual rates of bone loss (RBL) at the FN (p = 0.013), TH (p = 0.030) and TB (p = 0.00016). TB RBL was also higher for rs4774(C) homozygotes (p = 3.8E-5). This association was independent of weight change for FN and TB, but not for TH (Table 4). In addition, there was interaction between SNPs rs3087456*rs4774 for TB RBL (p = 8.7E-5, adjusted 0.002).

**Table 4:** *CIITA* association with rate of bone loss between age 75 and 80.

Site	Marker	Mean BMD (g/cm^2^)^1^	P-value^2^	Adjusted P-value^3^
Femoral neck	rs3087456	−1.398/−1.498/−2.096	0.013	0.036
Total Hip	rs3087456	−1.123/−1.333/−1.599	0.030	NS
Total Body	rs3087456	−0.299/−0.250/−0.356	0.00016	0.0011
Total Body	rs4774	−0.286/−0.266/−0.348	0.000038	0.00069

1 CC/CV/VV (c = common, v = variant allele).

2 Genotypic model.

3 Adjusted for weight change between age 75 and 80.

*CIITA* was not associated with body weight at baseline, but to weight change between age 75 and 80 (rs3087456, p = 0.016, genotypic model). Women with the rs3087456(G/G) genotype had a mean weight loss of 2.8 kg compared to 1.4 kg for women with the rs3087456(A/A) genotype.

Bone resorption markers were not associated with *CIITA* genotype, neither was blood biochemistry (CRP, ESR) or blood leukocyte count.

### Fracture

Among the OPRA participants, 51% (n = 507) had suffered at least one fracture between age 20 and 75, with no significant difference between the *CIITA* genotype groups. Incident fracture after age 75 was confirmed for 350 women (35%), resulting predominantly (99%) from low energy trauma. One or more osteoporotic fractures (at the hip, vertebra, distal radius or proximal humerus) occurred in 282 individuals (28%), of whom 110 (11%) had hip fracture.

The rs3087456(G) allele was protective against incident fracture overall, osteoporotic fractures and hip fracture (Table 5). The protective effect was significant even after correction for BMD and body weight at age 75 (Table 5).

**Table 5:** Association of *CIITA and CLEC16* polymorphisms with incident fractures between age 75 and 80 in the OPRA cohort.

Polymorphism	Genotype	Women without fracture (%)	Womenwithfracture (%)	p-value (adjusted)^1^	Odds Ratio(95% confidence interval)	
**Any fracture**						
***CIITA***_ rs3087456	AA	349 (61)	223 (39)	0.002		
	AG/GG	294 (71)	122 (29)	(0.005)	0.657 (0.491–0.879)	
**CLEC16A**_rs725613	TT	264 (61)	171 (39)	0.011		
	TG/GG	380 (69)	175 (32)	(0.013)	0.699 (0.526–0.928)	
**Hip fracture**						
**CIITA**_ rs3087456	AA	498 (87)	74 (13)	0.025		
	AG/GG	381 (92)	35(8)	(0.038)	0.626 (0.402–0.975)	
**CLEC16A**_rs725613	TT	392 (90)	43 (10)	NS		
	TG/GG	489 (88)	66 (12)	(NS)		
**Osteoporotic fracture** 2						
**CIITA**_ rs3087456	AA	395 (69)	177 (31)	0.021		
	AG/GG	315 (76)	101 (24)	(0.047)	0.732 (0.538–0.996)	
**CLEC16A**_rs725613	TT	297 (68)	138 (32)	0.027		
	TG/GG	415 (75)	140 (25)	(0.022)	0.705 (0.522–0.951)	

1 P-values calculated by χ^2^; adjusted for variables weight and TB BMD at 75y (Any fracture, Osteoporotic fracture) or weight and FN BMD at 75y (Hip fracture).

2 Osteoporotic fracture sites include hip, vertebrae, distal radius, proximal humerus.

### CLEC16A

LD between the *CLEC16A* SNPs was stronger between rs725613 and rs2903692 (D’ = 0.95, r^2^ = 0.84) than with rs6498169 (D’≥0.90, r^2^≥0.27). Three common (>10%) haplotypes were predicted (Supplementary Table S2).

### Bone Density and Bone Loss

Carriers of *CLEC16A* haplotype 1 (rs725613(T)/rs2903692(G)/rs6498169(A)) had lower LS BMD at baseline (−2%) and lower BMD at the 5 year follow-up at the FN (−1.3%) and TH (−1.5%) (Table 3). Women with genotype rs6498169(G/G) displayed lower TB BMD at 5-year follow-up (0.98 vs 1.00, p = 0.037) compared to the rs6498169(A/A) or (A/G) genotypes. For bone loss between age 75 and 80, women with the rs2903692(A/A) genotype displayed reduced TB RBL (−1.30 vs −1.51, p = 0.033).

The rs2903692 marker was also associated with QUS parameters, as carriers of the rare (A) allele had lower stiffness values (71.2 vs 72.0; p = 0.040) while BUA was lowest in the (G/A) heterozygotes (GG: 101.9, GA: 101.5, AA: 101.9; p = 0.045).

Carriers of *CLEC16A* haplotype 1 had higher blood leucocyte counts at 10 years compared to non-carriers (BLC 7.0 vs 6.3×10^9^/L, p = 0.002), while bone markers were not significantly associated.

### Fracture

Carriage of the rare *CLEC16A* rs725613(G) allele or *CLEC16A* haplotype 7 (rs725613(G)/rs2903692(A)/rs6498169(A)) was similarly protective against incident fracture overall, including after correction for TB BMD and body weight. The rs725613(G) allele was also protective of osteoporotic fractures, but not hip fracture specifically (Table 5).

### IFNG

The three markers in *IFNG* were in LD (D′ = 0.99, r^2^>0.45) and 3 common haplotypes were predicted (Supplementary Table S2).

### Bone Density and Bone Loss

None of the individual genotypes were associated with BMD at baseline or follow-up; however carriers of haplotype 4 (rs2069727(T)/rs2069718(T)/rs2069705(C)) had 1% lower baseline BMD at the hip. Interaction between rs2069727*rs2069705 was also observed at the lumbar spine (Table 3).

For the QUS phenotypes, there were small but significant differences in mean values. The variant alleles for all 3 *IFNG* SNPs, but not their derived haplotypes, were associated with increased BUA, the most significant being rs2069727(C) (p = 0.00003). Carriers of rs2069705(G) and *IFNG* haplotype 4 had lower SoS values (p = 0.003). The rs2069727(C) and rs2069718(C/T) genotypes were associated with slightly higher stiffness values with both a single-marker and interactive model (p = 0.033-0.05). There was no association between polymorphisms in the *IFNG* gene and bone loss, fracture, bone markers or blood biochemistry.

### Young Adult Women – the PEAK-25 Cohort

In young adult women representing peak bone mass, there was no association between *CIITA, CLEC16A or IFNG* SNPs and BMD. Small differences (<0.4%) between the genotype groups were observed between *CIITA* rs4774 and the QUS phenotype SoS (p = 0.031).

## Discussion

This study suggests that inflammatory genes play a role in regulating bone mass and bone loss and influence the risk of incident fractures in elderly women. Specifically, we report association between polymorphisms in the inflammatory genes *CIITA, CLEC16A* and *IFNG* with BMD, ultrasound parameters, annual rate of bone loss and incident fractures in 75-year old women followed for 10 years. These results support inflammation and, specifically, MHCII expression as key components in postmenopausal or senile osteoporosis.

The reported association of *CIITA* and *IFNG* polymorphisms to BMD in elderly women is contrasted by the absence of association in young adult women, which suggests differential effects of these genes across the life span. It also supports our hypothesis that low-grade systemic inflammatory processes are elements of normal ageing in women, possibly due to the link between estrogen and cytokine levels. Estrogen receptors are expressed on both lymphocytes and mononuclear cells [54], making them responsive to changes in estrogen levels. Estrogen deficiency leads to increased immune activation mediated by antigen presenting cells (APCs) and cytokines (IFNγ, IL-7 and transforming growth factor-β), resulting in increased TNFα production from activated T lymphocytes [24]. By increasing both the production and sensitivity to RANKL, TNFα has potent effects on osteoclasts [24]. Polymorphisms affecting immune activation processes could therefore have an impact during periods with generally higher pro-inflammatory profile, such as old age and illness. We interpret the discrepant results between the elderly and young adult women as a reflection of differences in duration of exposure to low-grade systemic inflammation. The potentially deleterious effects of inflammation should be more pronounced if the systemic inflammatory processes have continued for years. This could also explain why, despite numerous GWAS of osteoporosis related phenotypes [16]–[18], [55]–[71], these SNPs have not previously been identified; GWAS are not able to identify gene-environment interactions while the top 15 SNPs associated with BMD account for <3% of the variance of BMD [16].

Our observed results in elderly women - an association between functional polymorphisms in *CIITA*, BMD and fracture- is supported by previous reports evaluating the impact of the MHC2TA protein on molecular interactions and pathways. MHC2TA regulation of osteoclasts has been described in murine cells where it negatively regulated NFATc1 (nuclear factor of activated T-cells, cytoplasmic 1) and OSCAR by sequestering CBP/p300 from their promoter regions [72]. Decreased expression of *CIITA* would in this context lead to increased formation of osteoclasts. However, alternative mechanisms are also suggested since transgenic mice overexpressing *CIITA* display a hyper-osteoclastogenic phenotype and increased activation of signaling downstream of RANK (*E. Benasciutti and S. Cenci, personal communication).*

Based on our observations in this study and from earlier results from us and co-workers on expression levels [33], we postulate that the studied *CIITA* polymorphisms have osteoclast-stimulating effects that override the direct, inhibitory effect of IFNγ on osteoclasts [25]. Mechanistically this may be explained by estrogen deficiency after menopause, leading to increased production of IFNγ [24], [25] and an induction of *CIITA* expression in cells of the monocyte/macrophage lineage. The resulting MHC2TA protein acts as a transcriptional transactivator at MHCII promoters, and induces expression of MHCII molecules on antigen presenting cells [9]. The T cell pool then increases due to inhibited T cell apoptosis by the increased amount of IFNγ and more T cells become activated by binding MHCII molecules. The activated T cells produce TNF, which could stimulate osteoclast activity through induction of RANKL, and increase osteoclast number through stimulating cytokines. The increase in osteoclast activity could then lead to decreased BMD and increased risk of fracture. The observed protective effect from the *CIITA* rs3087456(G) allele is associated with lower expression of *CIITA* and MHCII [33], which could slow down the process of T cell- and osteoclast stimulation and result in higher BMD and reduced fracture risk.

In the present study, the inverse effect of *CIITA* variant alleles on rate of bone loss compared to BMD and fracture is however somewhat contradictory. Individuals carrying *CIITA* variant alleles (mainly *CIITA* rs3087456(G)) lost more bone mineral density between age 75 and 80, but had higher BMD at age 75, 80 and 85 and, importantly, were protected from incident fracture during this time. *CIITA* variant alleles thus had a net positive effect on bone density and bone strength in elderly women at all time-points, despite also being associated with a higher rate of bone loss. Gene effects on bone turnover were not reflected by the bone resorption markers S-CTX and S-TRACP5b, however, the low numbers with consistently high or low turnover may have masked such effects. Furthermore, markers indicate turnover (and the potential effect of inflammatory processes on it) only at the time of sample collection and may not be able to capture the long-term effects of inflammation.

In addition to *CIITA*, *CLEC16A* and to a lesser extent *INFG* were associated with bone mass phenotypes. However, the contribution to fracture risk is less clear; *CLEC16A* was associated with a significantly reduced fracture risk, although less than *CIITA*, while *INFG* was not. A potential explanation would be that the *CLEC16A* marker most strongly associated with fracture (rs725613), is located closest to, and is in weak LD with *CIITA*.

The strengths of the study include the comprehensive and extended evaluation of the women in the OPRA-cohort, including detailed fracture information and bone turnover markers. We also have the possibility to make direct comparisons of ageing effects through the large cohort of young adult women in the PEAK-25 cohort. In this study, the reported association with bone phenotypes and overall fracture risk in the elderly women support the relevance of inflammatory genes affecting MCHCII expression in the pathogenesis of osteoporosis.

The study has some limitations, notably the large number of tests performed. Taking this into consideration and applying a conservative Bonferroni correction, the results can be considered to fall short of p<0.05. Performing adjustment at least for the 8 markers tested, association with BMD is non-significant, whereas association of CIITA with total body RBL, fracture and hip fracture is still significant. Importantly however the direction of effect of the SNPs within each gene is consistent indicating biological plausibility for the observations and importantly, both cohorts are of sufficient size to be confident of detecting true genotype related differences in the phenotypes analysed.

Replication studies are needed in order to fully evaluate the effect of the studied SNPs on osteoporosis-related phenotypes. Thus, replication in a similarly aged cohort to OPRA is necessary to corroborate the findings. Secondly, routine biochemistry including leukocyte count, CRP and ESR as clinical indicators of inflammation was only available on all OPRA participants at age 85. The finding that *CLEC16A* haplotypes were associated with higher counts of blood leukocytes with a similar trend for single markers, suggests that it would have been interesting to have complete longitudinal data. Thirdly, longitudinal data for the young adult women would be potentially interesting (including follow-up extending into the perimenopausal years) in order to facilitate evaluation of the natural course of inflammatory influence with normal ageing and transition to an estrogen deplete state. In a study comparing levels of IFNγ in young, perimenopausal and elderly women, the levels of IFNγ increased at menopause compared to young women, but fell to even lower levels in the elderly [40]. These results strongly argue for testing the studied *CIITA* and *IFNG* polymorphisms in cohorts of perimenopausal women for comparison.

We conclude that expression-related polymorphisms in the inflammatory genes *CIITA* and *CLEC16A* are associated with BMD and fracture in elderly women. These findings illustrate the importance of inflammation and MHCII expression in particular in the pathogenesis of reduced bone strength in the elderly.

## Supporting Information

Table S1 **Allele and genotype frequencies for** ***CIITA*** **,** ***CLEC16A*** **and** ***IFNG*** **in the OPRA and PEAK25 cohorts.**
(DOC)

Table S2 ***CIITA*** **,** ***CLEC16A*** **and** ***IFNG*** **haplotype frequencies in the OPRA and PEAK25 cohorts.**
(DOC)

Table S3 **Weight and BMD at age 75, 80 and 85 years (y) in the OPRA cohort (n = 1003) and rate of bone loss (RBL) between 75 and 80 y.**
(DOC)
