# Supplementary material for: Polymorphisms in the Inflammatory Genes CIITA, CLEC16A and IFNG Influence BMD, Bone Loss and Fracture in Elderly Women
Source: PLoS One. 2012 Oct 25;7(10):e47964. doi: 10.1371/journal.pone.0047964 (PMC3485004; doi:10.1371/journal.pone.0047964)
Supplement: Table S1 — Allele and genotype frequencies for CIITA , CLEC16A and IFNG in the OPRA and PEAK25 cohorts. (DOC) [file pone.0047964.s001.doc]

**Supplementary information: Table S1**

Allele and genotype frequencies for *CIITA*, *CLEC16A* and *IFNG* in the OPRA and PEAK25 cohorts

|  | **Minor allele** | | **Genotype (CC / CV / VV)** | |
| --- | --- | --- | --- | --- |
| **Polymorphism** | **N^o^** | **Frequency** | **N^o^** | **Frequency** |
| **OPRA cohort (n=1003)** |  |  |  |  |
| CIITA_rs3087456(G) | 463 | 0.235 | 572 / 369 / 47 | 0.579 / 0.373 / 0.048 |
| CIITA_rs4774(C) | 610 | 0.311 | 477 / 404 / 103 | 0.485 / 0.411 / 0.105 |
| CLEC-16_rs725613(G) | 675 | 0.340 | 435 / 435 / 120 | 0.439 / 0.439 / 0.121 |
| CLEC-16_rs2903692(A) | 640 | 0.322 | 458 / 430 / 105 | 0.461 / 0.433 / 0.106 |
| CLEC-16_rs6498169(G) | 791 | 0.396 | 361 / 483 / 154 | 0.362 / 0.484 / 0.154 |
| INFG_rs2069727(C) | 936 | 0.468 | 298 / 470 / 233 | 0.298 / 0.470 / 0.233 |
| INFG_rs2069718(T) | 820 | 0.418 | 325 / 494 / 163 | 0.331 / 0.503 / 0.166 |
| IFNG_rs2069705(C) | 691 | 0.374 | 425 / 451 / 120 | 0.427 / 0.453 / 0.120 |
|  |  |  |  |  |
| **PEAK25 cohort (n=999)** |  |  |  |  |
| CIITA_rs3087456(G) | 533 | 0.270 | 530 / 381 / 76 | 0.537 / 0.386 / 0.077 |
| CIITA_rs4774(C) | 585 | 0.301 | 476 / 403 / 91 | 0.491 / 0.415 / 0.094 |
| CLEC16_rs725613(G) | 671 | 0.341 | 428 / 441 / 115 | 0.435 / 0.448 / 0.117 |
| CLEC16_rs2903692(A) | 641 | 0.324 | 452 / 431 / 105 | 0.457 / 0.436 / 0.106 |
| CLEC16_rs6498169(G) | 783 | 0.393 | 370 / 469 / 157 | 0.371 / 0.471 / 0.158 |
| INFG_rs2069727(C) | 910 | 0.456 | 299 / 488 / 211 | 0.300 / 0.489 / 0.211 |
| INFG_rs2069718(T) | 847 | 0.435 | 311 / 479 / 184 | 0.319 / 0.492 / 0.189 |
| IFNG_rs2069705(C) | 651 | 0.334 | 436 / 425 / 113 | 0.448 / 0.436 / 0.116 |

C=common allele, V=variant allele
