# Supplementary material for: Polymorphisms in the Inflammatory Genes CIITA, CLEC16A and IFNG Influence BMD, Bone Loss and Fracture in Elderly Women
Source: PLoS One. 2012 Oct 25;7(10):e47964. doi: 10.1371/journal.pone.0047964 (PMC3485004; doi:10.1371/journal.pone.0047964)
Supplement: Table S2 — CIITA , CLEC16A and IFNG haplotype frequencies in the OPRA and PEAK25 cohorts. (DOC) [file pone.0047964.s002.doc]

**Supplementary information: Table S2**

***CIITA*, *CLEC16A* and *IFNG* haplotype frequencies in the OPRA and PEAK25 cohorts**

| **Gene** | **Haplotype Alleles*** | **OPRA (%)** | **PEAK25 (%)** |
| --- | --- | --- | --- |
| ***CIITA*** | AG | 50.3 | 48.4 |
|  | AC | 26.2 | 24.6 |
|  | GG | 18.7 | 21.5 |
|  |  |  |  |
| ***CLEC16A*** | TGA | 26.8 | 26.9 |
|  | TGG | 38.5 | 37.9 |
|  | GAA | 31.8 | 31.3 |
|  |  |  |  |
| ***IFNG*** | TCT | 11.3 | 11.1 |
|  | TTC | 34.6 | 33.4 |
|  | CCT | 46.6 | 45.5 |

*Haplotypes for *CIITA* are reported in the order: rs3087456 (A/G), rs4774 (G/C); Haplotypes for *CLEC16A* are reported in the order: rs725613 (T/G), rs2903692 (G/A), rs6498169 (A/G); Haplotypes for *IFNG* are reported in the order: rs2069727 (T/C), rs2069718 (C/T), rs2069705 (T/C)
