# Supplementary material for: Polymorphisms in the Inflammatory Genes CIITA, CLEC16A and IFNG Influence BMD, Bone Loss and Fracture in Elderly Women
Source: PLoS One. 2012 Oct 25;7(10):e47964. doi: 10.1371/journal.pone.0047964 (PMC3485004; doi:10.1371/journal.pone.0047964)
Supplement: Table S3 — Weight and BMD at age 75, 80 and 85 years (y) in the OPRA cohort (n = 1003) and rate of bone loss (RBL) between 75 and 80 y. (DOC) [file pone.0047964.s003.doc]

**Supplementary Table S3**

Weight and BMD at age 75, 80 and 85 years (y) in the OPRA cohort (n=1003) and rate of bone loss (RBL) between 75 and 80 years.

| **Variable** | **Mean** | **SD** | **(Range)** | **N^o^** |
| --- | --- | --- | --- | --- |
|  |  |  |  |  |
| Weight 75 y (kg) | 67.8 | ±11.5 | (41−110) | 1003 |
| Weight 80 y (kg) | 66.2 | ±11.5 | (34−105) | 702 |
| Weight 85 y (kg) | 63.8 | ±10.7 | (39-102) | 376 |
|  |  |  |  |  |
| **BMD (g/cm^2^)** |  |  |  |  |
| Total body 75 y | 1.007 | ±0.097 | (0.718−1.422) | 904 |
| Femoral neck 75 y | 0.748 | ±0.130 | (0.153−1.230) | 924 |
| Total hip 75 y | 0.849 | ±0.149 | (0.498−1.416) | 903 |
| Lumbar spine 75 y | 0.993 | ±0.195 | (0.518−1.855) | 946 |
|  |  |  |  |  |
| Total body 80 y | 0.997 | ±0.098 | (0.724−1.322) | 668 |
| Femoral neck 80 y | 0.713 | ±0.128 | (0.152−1.475) | 678 |
| Total hip 80 y | 0.800 | ±0.134 | (0.261−1.393) | 676 |
|  |  |  |  |  |
| Total body 85 y | 0.992 | ±0.103 | (0.684−1.383) | 371 |
| Femoral neck 85 y | 0.689 | ±0.137 | (0.145−1.175) | 361 |
| Total hip 85 y | 0.767 | ±0.138 | (0.217−1.277) | 361 |
|  |  |  |  |  |
| **RBL (%)** |  |  |  |  |
| Total body RBL | -0.282 | ±0.643 | (-3.09−1.80) | 630 |
| Femoral neck RBL | -1.472 | ±2.165 | (-13.89−7.70) | 655 |
| Total hip RBL | -1.230 | ±1.868 | (-10.39−6.33) | 638 |
